# After the Harrison Law, What?

**Published:** 1915-05

**Authors:** 


### After The Harrison Law, What?

The license to practice medicine involves arduous duties, heavy responsibilities, and certain privileges, not intended to be of a personal nature, but obviously susceptible to abuse. One of these privileges is easy access to poisons and means of administering them. This privilege has been limited to some degree by law, but not so as to impose any special hardship upon the medical profession or to render the misuse of poisons, either by the profession if it chose to become Medici in Italian as well as Latin, or by the laity especially difficult. When we consider that almost any chemic substance may be employed, in suitable dose and by suitable methods, as a poison, it is plain that legislation of this nature is bound to be inefficient—not to mention the substitution of other than chemic means for suicidal or homicidal ends.

At present, the profession of several states is embarrassed by the inconsistencies of both state and national laws, in regard to certain narcotic drugs. Not to dwell either on the general principles of retro-active legislation, the constitutionality of invasion of local fields by national law, and the practical difficulties of obeying two masters, state and national, in submission to the law without these general questions, it may not be out of place to point out certain other opportunities for special regulation and license.

The privacy of a professional consultation of any sort, legal, clerical or medical, obviously may be and sometimes actually is abused across sex lines. Thus, opportunity is afforded for inquisition as to the personal conduct of the physician, for the preparation of special blanks for reporting such confidences, for the establishment of a board of chaperones, appointed by civil service or other methods.

Hypnotism is still occasionally employed, though somewhat unfashionable at present, at least under this name. Here is a chance for further special legislation, blanks, boards of registration, etc.

Surgeons still claim that patients are referred too late for operations, while internists sometimes retaliate with charges of unnecessary operations or operations by incompetent hands. Here is an indication for a special examination and license for surgical practice—a good thing in its way but presenting difficulties in emergent cases in inaccessible regions, not to mention the equal importance of competence for chemic and bacteriologic, diagnostic and medical therapeutic problems. In addition, analogy to the Harrison law, would require a sort of referee system to determine the question of advisability of operation, some kind of judicial proceeding to commit the unwilling patient for operation, as the unwilling insane patient is committed to an asylum—and more blanks.

It can scarcely be disputed that some of the greatest sins of omission and commission in therapeutics are perpetrated in diet. At least, this is one of the branches of therapeutics along which the most radical disagreements are expressed. The administration of the pure food and drug laws could be greatly extended, especially in its personnel—always a desirable practical feature of legislation—and further license, report blanks and supervision would be in order.

Cathartic drugs, though largely used by the laity and dispensed over the counter of the drug store without much opposition from the medical profession are often ultimately harmful and, in general, require a careful selection, dosage and timing of administration to produce the desired results. To a much greater degree of perfection than either acute poisons, abortifacient drugs, narcotics and habit forming drugs, are cathartics susceptible of accurate listing. Thus, it would be comparatively easy to make a list of cathartics, to be reported on special blanks, to be preserved for five years—this period of time being really necessary for the development of ultimately harmful results of serious degree—and a dollar or two a year could be assessed against physicians and druggists for assisting patients to secure these drugs.

Water, ordinary, distilled and mineral, charged or still, is susceptible of misuse. Death, local and somatic, may even be caused by the inordinate application of distilled water. The obvious opportunity exists for further special legislation.

We forego the pleasure of extending this list further. There is no therapeutic agent, drug or imponderable, that is not susceptible of misuse, intentional or ignorant, no field of practice in which diagnosis and therapeutics do not demand both skill and conscience, no case in which the extra-professional influence of the physician may not be exercised for great good or great evil. The confidential relations necessary in various professions and businesses are liable to be abused. In no other profession is the intimacy and privacy of these relations so inevitable as in the case of the medical profession. Along with responsibility and ability to do good, lies the power to do harm. There is only one efficient safe-guard—the skill, and good conduct of the physician himself. No special law can protect the victim if this is absent. We can go further from actual experience in regard to the abortion evil; no special system of safe guards can have any great degree of efficiency either as a deterrent of crime or to detect and punish it. Special restrictions of this or that item of practice tend to violate the confidence of the professional relation, to hamper the physician in case of emergency, to distract his attention from the main issue, the relief and cure of disease. When all is said and done, the fact remains that the physician is either competent and conscientious or he is not. If he is false and unreliable in one item of practice, he is so in all. We believe, therefore, in one, general license to ractice medicine, permanent except for violations of its general provisions. Having granted this, the detection and punishment of incompetence and of willful criminality should follow the same general lines as for any other violation of law and should not be confused with a mass of special legislation whose efficiency is limited almost entirely to the detection and punishment of self-created, technical and essentially innocent infractions.

